# Appropriate Immediate Dentin Sealing to Improve the Bonding of CAD/CAM Ceramic Crown Restorations

**DOI:** 10.3390/polym14214541

**Published:** 2022-10-26

**Authors:** Miwa Nakazawa, Masahiko Maeno, Mei Komoto, Yoichiro Nara

**Affiliations:** Department of Adhesive Dentistry, School of Life Dentistry at Tokyo, The Nippon Dental University, Tokyo 102-8159, Japan

**Keywords:** immediate dentin sealing, bonding performance, CAD/CAM restoration, ceramic crown, cyclic loading, micro-tensile bond strength, Weibull analysis

## Abstract

This study aimed to use quantitative and qualitative evaluations based on micro-tensile bond strength (μTBS) to clarify the appropriate immediate dentin sealing (IDS) approach for improving the bonding of CAD/CAM ceramic crown restorations. Forty-eight extracted human molars were prepared to obtain standardized abutment specimens and divided into three groups: no IDS (group C: control), IDS performed by a single application of an all-in-one adhesive system (group A), and IDS performed by the combined application of an adhesive system and a flowable resin composite (group F). All specimens were restored with a ceramic crown fabricated by a chair-side CAD/CAM system and were divided into no-stress and stressed groups. After cyclic loading (78.5 N; total, 3 × 105 cycles; 90 cycles/min) on the specimens in the stressed group, all specimens were sectioned. The μTBS values for the occlusal and mesioaxial walls were measured (*n* = 16) and analyzed statistically. The quantitative bonding performance of groups A and F were superior to that of group C, regardless of the cyclic loading and abutment wall conditions. Group F showed the maximum bond strength and the highest bond durability in the qualitative bonding performance even under the cyclic loading condition simulating clinical mastication.

## 1. Introduction

The rapid evolution of CAD/CAM technology has had a dramatic impact on the fields of prosthodontics and restorative dentistry [1]. In particular, chair-side CAD/CAM systems have gained popularity globally because they can be used to fabricate various restorations with lower chair time in comparison with the conventional restoration method while offering acceptable marginal adaptation, better clinical longevity [2], high success rate, clinically acceptable wear rate, and good color stability [3].

Immediate dentin sealing (IDS) is known to be useful for improving the bonding state and reduce both gap formation and postoperative dentin sensitivity of metal-free CAD/CAM restorations [4,5,6,7,8,9,10]. IDS can be performed by the single application of an all-in-one adhesive system [4,6,11,12,13,14] or by the combined application of both the adhesive system and a flowable resin composite [4,5,14,15]. Although IDS with an adhesive system alone has been reported to improve the bond strength of the resin cement to dentin [16], the combined approach yields improved bonding of the CAD/CAM ceramic restoration due to the stress-breaking function of the flowable resin composite [10]. Moreover, IDS with both materials provides insulation against thermal stimulation [15]. IDS is also known to improve the adaptation of CAD/CAM ceramic crown restorations [17]. Considering these advantages, IDS of either type may be beneficial for metal-free indirect restorations.

Previous studies have examined the bonding performance of IDS without any stress or under thermal cycling stress conditions [6,11,18,19]. Only Feitosa et al. [20] evaluated bonding under a cyclic loading condition. However, their study was conducted using flattened dentin surface specimens. Thus, no previous study has clarified the bonding of ceramic restorations with different IDS conditions under cyclic loading conditions simulating the intraoral environment. An investigation of the difference in bonding between the occlusal surface of the abutment where the cyclic loading stress is vertically applied and the axial surface where the shear stress occurs with cyclic loading is important for clarifying the bonding behavior of CAD/CAM ceramic crown restorations.

Bond strength is a typical indicator of bonding. The micro-tensile bond strength (μTBS) test proposed by Sano et al. [21] can measure changes in bond strength under the influence of various clinical factors, such as material properties and thermal and mechanical stresses. Thus, investigations based on μTBS can serve as quantitative evaluations of bonding. On the other hand, Weibull analysis [22] is an effective approach to assessing measured bond strength [23], and the results obtained from this analysis can provide a qualitative evaluation of bonding. The International Organization for Standardization (ISO) [23] states that the Weibull stress values for 10% and 90% failure probability level (PF10 and PF90) are convenient measures to characterize the strength of a bond.

To clarify the IDS-induced improvement in the bonding of CAD/CAM ceramic crown restorations, both quantitative and qualitative evaluations were conducted for this study based on μTBS under conditions with and without cyclic loading (78.5 N; total, 3 × 105 cycles; 90 cycles/min; in circulating water at 37 °C) and the difference between the occlusal and axial abutment walls. The null hypotheses of this study were as follows:

1. The type of IDS does not influence the bonding of CAD/CAM ceramic crown restorations.
2. The presence of cyclic loading does not affect the bonding.
3. Bonding does not differ between the occlusal and axial abutment walls.

## 2. Materials and Methods

### 2.1. Experimental Material

Table 1 lists the product name, composition, lot number, and manufacturer for each material used in this study. An all-in-one adhesive system (Clearfil Universal Bond Quick; Kuraray Noritake Dental, Tokyo, Japan) was used alone or in combination with a flowable resin composite (Clearfil Majesty ES Flow; Kuraray Noritake Dental) for IDS treatment. An adhesive resin cement (PANAVIA SA Cement Universal; Kuraray Noritake Dental) that demonstrated self-adhesive ability not only to dentin but also to various restorative materials (e.g., ceramics, resin composite, metal alloy) was used for the luting of CAD/CAM ceramic restorations. The pretreatment of the inner surface of the restorative prior to luting was performed using 9.5% hydrofluoric acid (Porcelain Etchant; BISCO, Schaumburg, IL, USA). A typical chair-side CAD/CAM system (CEREC AC Omnicam SW v4.5 and CEREC MC XL; Dentsply Sirona, York, PA, USA) was used for the scanning, designing, and fabrication of the ceramic crown. A feldspathic ceramic block (VITABLOCS Mark II; VITA Zahnfabrik, Bad Säckingen, Germany) was selected as the material for the CAD/CAM crown. All light irradiation procedures were performed with a light-emitting diode (LED) curing source (G-Light Prima II, GC, Tokyo, Japan) in the normal mode (870 mW/cm^2^) after the confirmation of the light intensity prior to each irradiation.

### 2.2. Tooth Selection and Experimental Procedures

**Figure 1:**
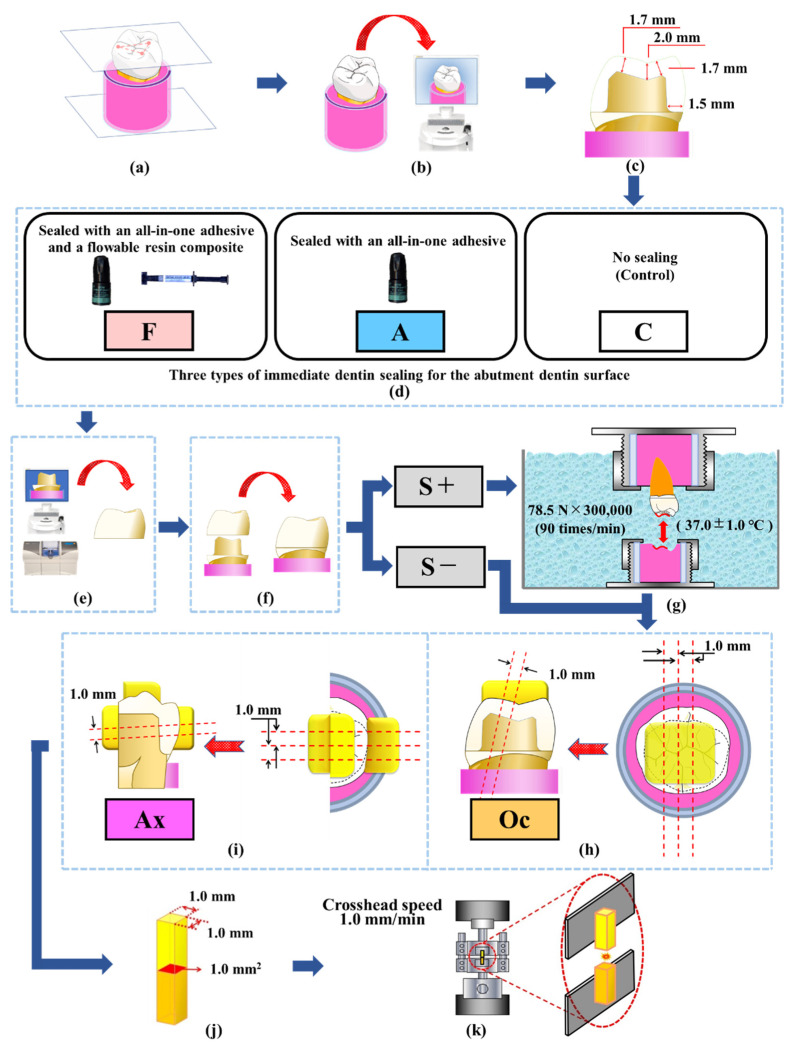
Flowchart of the experimental procedure. (**a**): Standardized tooth embedding, (**b**): Scanning of intact coronal form, (**c**): Standardized abutment preparation, (**e**): Fabrication of CAD/CAM ceramic crown, (**f**): Luting of fabricated crown, (**g**): Cyclic loading of 78.5 N; total, 3 × 105 cycles; 90 cycles/min in circulating water at 37 °C, (**h**–**j**): Sectioning in order to obtain the micro-tensile bond strength (μTBS) beam specimen, (**k**): μTBS measurement at a crosshead speed of 1.0 mm/min.

This use of extracted human teeth in this study was approved by the ethics committee of Nippon Dental University, School of Life Dentistry at Tokyo (Approval number: NDU-T2019-32). A total of 48 sound mandibular first molars of similar size and color-tone that had been stored in 0.1% thymol solution at 23 ± 2 °C for less than one year after extraction were selected for this study. Figure 1 presents a flowchart of the experimental procedures.

Each tooth was embedded in a standardized cylindrical plastic mold filled with an acrylic resin (PROVINICE; Shofu, Kyoto, Japan) 1 mm below from the lowest point of the anatomical buccocervical line, which was established as a plane set with three apices of the mesiobuccal, distobuccal, and mesiolingual cusps parallel to the base plane of the mold (Figure 1a). The intact coronal form of each embedded tooth was scanned with the intraoral scanner of the CAD/CAM system to reproduce the original crown shape onto each fabricated ceramic crown (Figure 1b).

For standardized abutment preparation, a straight cylinder diamond bur (FG211; ISO 110 070 014; average grain size: 100 µm; Shofu) was used for occlusal preparation, and a round-end diamond bur (FG107RD; ISO 198 090 023; average grain diameter: 100 µm; Shofu) was used for the axial wall and finishing line preparation. The two types of diamond burs were replaced after every five abutment preparations. The standardized abutment of the tooth specimen was prepared with a custom-made abutment duplicator (Tokyo Giken, Tokyo, Japan) on the basis of the proposed dimensions for the CAD/CAM ceramic crown restoration [24].

The tooth substance was removed to a depth of 2.0 mm from the central fossa and 1.7 mm from the inner inclined surfaces of the cusp by using the straight cylinder diamond bur. Next, the axial wall with a rounded shoulder of 1.5 mm width was prepared with the round-end diamond bur (Figure 1c). Subsequently, all abutment specimens were randomly divided into three groups based on the type of IDS performed: no IDS (group C: control), IDS with single application of an all-in-one adhesive system (group A), or IDS with combined application of the adhesive system and a flowable resin composite (group F).

In group A, the adhesive system was applied on the prepared dentin surface of specimens and gently air-dried for 5 s, followed by light-curing for 10 s. In group F, approximately 50 mg of the flowable resin composite was applied with a small brush on the prepared dentin surface to achieve an average sealing thickness of 85 µm, which was followed by the same surface treatment as in group A and light-curing for 20 s. The unpolymerized superficial layer of the IDS specimens in both A and F were sufficiently wiped and removed by a cotton pellet soaked in 70% ethanol (Figure 1d).

All abutment specimens of the three types of IDS were scanned with the intra-oral scanner of the CAD/CAM system in accordance with the manufacturer’s instructions. Each CAD/CAM ceramic crown was designed using both the duplicate function of the system and the digital data for the intact coronal form, and then fabricated by the system (Figure 1e). Before luting, each inner surface of the fabricated crown was etched with 9.5% hydrofluoric acid for 90 s, rinsed, and air-dried. The adhesive resin cement was applied to the etched inner surface of the crowns, and the crowns were pressed onto the abutment under a force of 8.8 N for 1 min. After the removal of excess cement followed by tuck-cure, each specimen was light-irradiated from the occlusal, mesial, distal, buccal, and lingual sides by the LED curing source for 10 s each, for a total irradiation period of 50 s (Figure 1f). The marginal region of every restored specimen was polished with a series of polishing discs (Sof-Lex XT; 3M, St. Paul, MN, USA) and then stored in 37 °C water for 1 h (Figure 1i).

### 2.3. Cyclic Loading and Micro-Tensile Bond Strength Test

All specimens restored by the three types of IDS were randomly divided into no stress (S−) and stressed (S+) conditions. For the S+ condition, an opposing object was prepared individually using a hard acrylic resin (PROVINICE, Shofu) against the inner and the outer inclined surfaces of the functional cusps and the inner inclined surface of non-functional cusps. Then, each restored specimen of the S+ condition was subjected to cyclic loading (78.5 N; total, 3 × 10^5^ cycles; 90 cycles/min) in circulating water at 37 °C by a custom-made multifunctional apparatus (Tokyo Giken, Tokyo, Japan) [8,9,10] (Figure 1g).

Subsequently, additional resin composite (Clearfil AP-X; Kuraray Noritake Dental) build-up with an all-in-one adhesive system (Clearfil Universal Bond Quick, Kuraray Noritake Dental) was performed on the occlusal surface of all restored specimens to prepare the μTBS beam specimen, regardless of stress condition. Two slab specimens of 1.0 mm thickness, which included the bonded dentin surface on the inner inclined surface of the functional cusps (Oc), were obtained by sectioning three times along the buccolingual dimension by using a diamond wire saw (DMS3400; Meiwa fosis, Tokyo, Japan) (Figure 1h). Similarly, two slab specimens of 1.0 mm thickness, which included the dentin surface on the mesioaxial wall (Ax) of the prepared abutment, were obtained by the sections along the mesiodistal dimension after additional resin composite build-up on both the mesial proximal surface and the buccolingual sectioned dentin surface of the remaining cut specimen (Figure 1i).

Slab specimens of 1.0 mm thickness were trimmed to standardized beam-shaped specimens (bonded area, 1.0 mm × 1.0 mm) with a diamond wire saw (Figure 1j). Then, the µTBS of each beam specimen was measured at a crosshead speed of 1.0 mm/min using a universal testing machine (Autograph AG-1; Shimadzu, Kyoto, Japan) (Figure 1k). When the specimens failed to obtain the beam specimen during preparation (pretesting failure (ptf)), the value of the specimen was replaced with a random value between zero and the lowest µTBS measured for the respective group [25].

### 2.4. Statistical Analysis

The obtained µTBS values were analyzed with the Kruskal–Wallis test, the Steel–Dwass test, and the Mann–Whitney U test. In addition, three typical Weibull parameters based on µTBS, namely, Weibull modulus (Wm) and Weibull stress at a probability of failure of 10% and 90% (PF10/PF90), were qualitatively analyzed to evaluate the bonding reliability and durability. Fracture mode distribution was analyzed with the chi-square test [26]. All statistical analyses were carried out using spreadsheet software (Excel 2016 for Windows; Microsoft, Redmond, WA, USA) with the level of significance set at 5%.

### 2.5. Fracture Mode Observation

The fractured surface of each post µTBS test specimen was observed under an optical microscope (Measurescope MM-11; Nikon, Tokyo, Japan) at 200× magnification. In addition, the typical specimen in each experimental condition was observed to confirm the fracture mode composition and the structure of each fractured surface using a scanning electron microscope (SEM; JSE-IT200; Hitachi, Tokyo, Japan) with an accelerating voltage of 5.0 kV after mounting to specimen stub using conductive tape and osmium coating (approximate 10 nm thickness) on the abutment-side fractured surface.

## 3. Results

### 3.1. Differences in the Micro-Tensile Bond Strength on the Occlusal and Axial Walls among the Three Types of Immediate Dentin Sealing with and without Cyclic Loading

**Figure 2:**
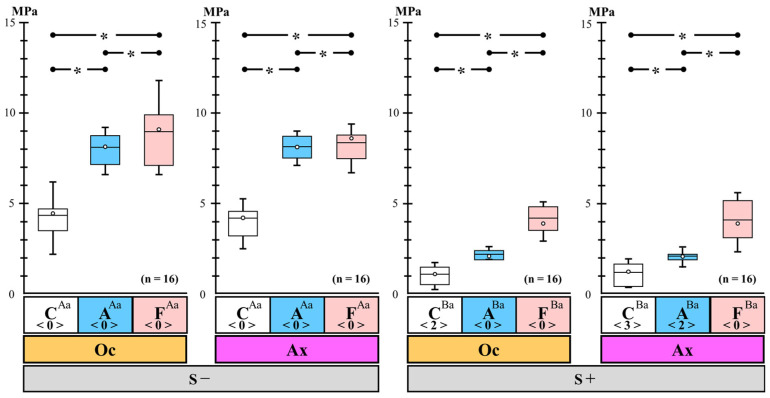
Differences in the micro-tensile bond strength on the occlusal and axial walls among the three types of immediate dentin sealing with and without cyclic loading. The box indicates the spread of the data between the first and third quartiles. The central horizontal line and the circle show the median and the mean, respectively. Maximum and minimum values are indicated by whiskers. The number of pretesting failure specimens is indicated by angle brackets. The asterisks (*) among the three types of immediate dentin sealing indicate statistically significant differences at *p* < 0.05. Different uppercase letters between two conditions with and without cyclic loading in the same immediate dentin sealing and in the same abutment wall indicate statistically significant differences at *p* < 0.05. Different lowercase letters between two different abutment walls in the same immediate dentin sealing and in the same cyclic loading condition indicate statistically significant differences at *p* < 0.05.

The differences in µTBS between the Oc and Ax groups among the three types of IDS with and without cyclic loading are shown in Figure 2. The micro-tensile bond strength test results are shown in Table 2. The μTBS values in groups A and F were significantly greater than that in group C, regardless of the cyclic loading and abutment wall conditions. The μTBS in group F showed the maximum bond strength in comparison with groups A and C and was significantly greater than the value for group A, irrespective of cyclic loading and abutment wall conditions. Thus, the quantitative bonding performance of groups A and F with IDS was superior to that of group C (control), regardless of the cyclic loading and abutment wall conditions. In particular, group F showed the maximum bond strength.

The μTBS values under the S+ condition were significantly smaller than the values under the S− condition, regardless of IDS and abutment wall conditions. Furthermore, for the S+ group, ptf specimens were observed in both group C (Oc: 2, Ax: 3) and group A (Ax: 2). Thus, cyclic loading decreased the initial bond strength of CAD/CAM ceramic crown restorations, regardless of the IDS and abutment wall conditions. Moreover, μTBS in the Oc and Ax groups did not differ significantly regardless of cyclic loading and IDS. Thus, the internal bond strength of CAD/CAM ceramic crown restorations was not influenced by differences in abutment wall conditions.

### 3.2. Differences in Weibull Parameters for the Occlusal and Axial Walls among the Three Types of Immediate Dentin Sealing with and without Cyclic Loading

**Figure 3:**
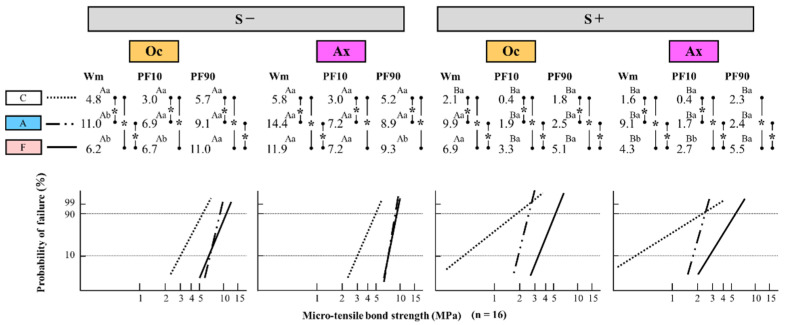
Differences in Weibull parameters for on the occlusal and axial walls among the three types of immediate dentin sealing with and without cyclic loading. Wm: Weibull modulus; PF10: Weibull stress (MPa) for a 10% failure probability; PF90: Weibull stress (MPa) for a 90% failure probability. The asterisks (*) among the three types of immediate dentin sealing indicate statistically significant differences at *p* < 0.05. Different uppercase letters between two conditions with and without cyclic loading in the same immediate dentin sealing and in the same abutment wall indicate statistically significant differences at *p* < 0.05. Different lowercase letters between two different abutment walls in the same immediate dentin sealing and in the same cyclic loading condition indicate statistically significant differences at *p* < 0.05.

The differences in the Weibull parameters Wm and PF10/PF90 in the Oc and Ax walls among the three types of IDS with and without cyclic loading are shown in Figure 3. The Weibull parameter values of each experimental condition is shown in Table 3. For the three types of IDS, Wm, PF10, and PF90 in groups A and F, except in one condition (S+/Ax/PF90), were significantly greater than the values in group C, regardless of the cyclic loading and abutment wall conditions. In addition, group C showed the minimum value in both conditions. Thus, the qualitative bonding performance in groups A and F with IDS was superior to that in group C (control) regardless of the conditions. Moreover, Wm in group A was significantly greater than that in group F irrespective of the conditions. Thus, group A showed the most excellent bonding reliability among the three types of IDS, regardless of the cyclic loading and abutment wall conditions.

The PF10/PF90 assessments showed no significant differences under the S− condition between groups F and A except in one condition (Oc/PF90). The values under the S+ condition in group F were significantly greater than the values in group A, irrespective of the abutment wall condition. For the cyclic loading condition, the bonding durability in group F showed the most outstanding state among the three groups, regardless of the abutment wall conditions. Moreover, in cyclic loading, the Weibull parameters under the S+ condition were significantly smaller than those under the S− condition, except two Wm values in Oc/A and F conditions, regardless of the IDS and abutment wall conditions. Thus, cyclic loading clearly tended to cause the deterioration of qualitative bonding performance, regardless of IDS and abutment wall conditions.

In assessments of the abutment wall, Wm and PF10 for Oc under the S− condition were similar to or significantly smaller than for Ax. On the other hand, the corresponding values for Ax under the S+ condition were similar to or significantly smaller than the Oc values. For PF90, the Ax values under the S− condition were similar to or significantly smaller than the Oc values, although no significant differences were observed between the values of the two abutment walls under the S+ condition. Thus, the bonding reliability of the Ax wall tended to be superior to that of the Oc wall, and the bonding durability varied with cyclic loading and the abutment wall condition.

### 3.3. Fracture Mode Distribution of the Post-Bond Test Specimens

The distribution of fracture mode in the post-bond test specimens is shown in Table 4.

The significant differences in the distribution of fracture mode among three types of IDS were indicated by chi-square test. Every fractured surface was composed of combinations of interfacial fractures at the interface between the restorative and resin cement (Ri), cohesive fractures within the resin cement (Cc), and interfacial fractures at the interface between the resin cement and abutment side surface (i.e., dentin, adhesive, or flowable resin surfaces) (Ai). Cc was observed on every fractured surface, regardless of the IDS, cyclic loading, and abutment wall conditions. For the S− condition, the fracture surfaces of groups C and A mainly showed Cc + Ai mixed fractures (91.6% and 65.6% respectively), regardless of abutment wall condition. In addition, the numbers of both Ri + Cc and Cc + Ai mixed fractures in group F were almost equivalent. For the S+ condition, similar to the S− condition, the fracture surfaces of groups C and A primarily indicated Cc + Ai mixed fractures (84.4% and 62.5% respectively), whereas the surfaces of group F showed Ri + Cc mixed fractures (78.1%). No ptf specimen was recognized in the S− condition, but ptf occurred in groups C (Oc: 2, Ax: 3) and A (Ax: 2) under the S+ condition. Thus, cyclic loading may damage the resin cement itself or the interface between resin cement and abutment wall in groups C and A, and probably attack the resin cement itself or the interface between the restorative and the resin cement in group F.

**Figure 4:**
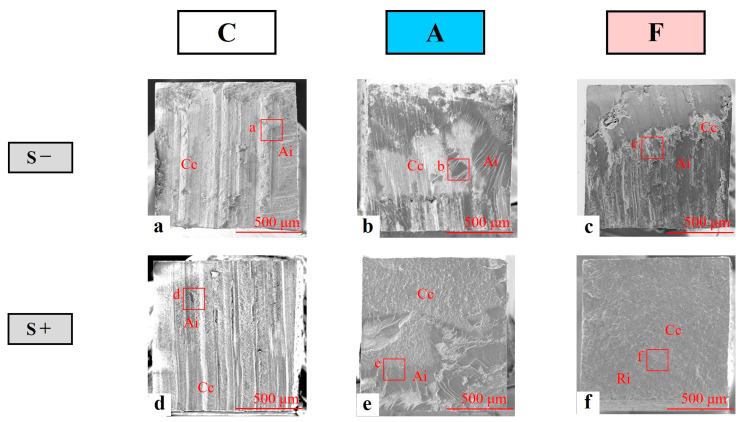
Representative scanning electron microscopy images of the dentin-side surface of the post-test specimens on the occlusal wall (50× magnification). Interfacial fracture occurred at the interface between the restorative and resin cement (Ri), cohesive fracture occurred within the resin cement (Cc), and interfacial fracture occurred at the interface between the resin cement and the abutment-side surface (i.e., dentin, adhesive, or flowable resin) (Ai).

Representative SEM images of the Oc wall and the dentin-side surfaces of the post-bond test specimens in the S− (a, b, c) and S+ (d, e, f) conditions are shown in Figure 4 (50× magnification) and 5 (500× magnification).

**Figure 5:**
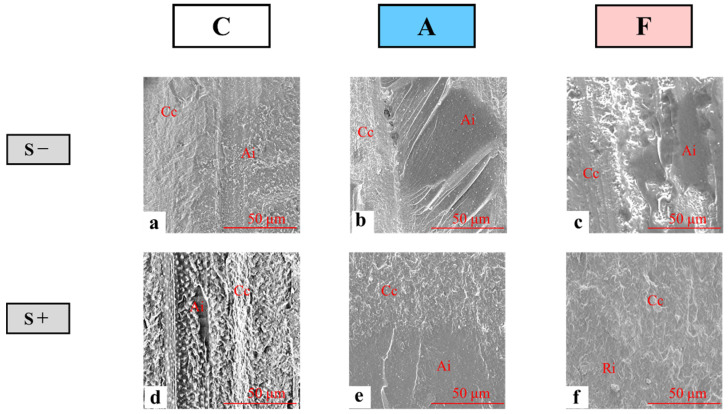
Representative scanning electron microscopy images of the dentin-side surface of the post-test specimens on the occlusal wall (500× magnification). Interfacial fracture occurred at the interface between the restorative and resin cement (Ri); cohesive fracture occurred within the resin cement (Cc), and interfacial fracture occurred at the interface between the resin cement and the abutment-side surface (i.e., dentin, adhesive, or flowable resin) (Ai).

At low magnification, group C specimens show wavy Cc surfaces and moderately coarse Ai surfaces regardless of cyclic loading (Figure 4a,d). At high magnification, the fractured surface displayed coarse Cc surfaces and moderately coarse Ai surfaces (Figure 5a,d). In particular, for the S+ condition, the existence of dentinal tubules was partially recognized on the surface of the specimens (Figure 5d). Low-magnification images of group A specimens with a post-bond test surface showed coarse Cc surfaces and smooth Ai surfaces with minute corrugation regardless of cyclic loading (Figure 4b,e), while high-magnification images showed coarse Cc surfaces and fractured adhesive surfaces with a wrinkled structure (Figure 5b,e). Low-magnification images of group F specimens under the S− condition show the Cc surface and flat Ai surface between the resin cement and flowable resin composite (Figure 4c), while high-magnification images show the fractured surface with a coarse Cc surface and a slightly wavy flowable resin surface (Figure 5c). On the other hand, low-magnification images of group F specimens under the S+ condition showed the Cc surface and a slightly coarse Ri surface (Figure 4f), while high-magnification images showed the post-bond test surface with a coarse Cc surface and irregularly wavy Ri cement surface (Figure 5f).

## 4. Discussion

### 4.1. Differences in the Micro-Tensile Bond Strength for the Occlusal and Axial Walls among the Three Types of Immediate Dentin Sealing with and without Cyclic Loading

To clarify the influence of differences in IDS on adhesion, a typical self-adhesive resin cement without any pretreatment was employed in this study. Thus, the bond strength in group C reflected the quantitative adhesion of the resin cement to both the abutment dentin surface and the internal surface of the CAD/CAM ceramic crown. On the other hand, the bond strengths in groups A and F reflected the quantitative bonding performance of IDS to the abutment dentin surface. IDS, which can be performed using a single application of the adhesive system or combined application of both the adhesive system and a flowable resin composite, has been shown to be effective in improving the dentin bond strength of metal-free indirect restorations [4,6,8,9,10,11,27]. Therefore, IDS application in groups A and F probably contributed to the higher bond strength than that in group C (control), which was cemented with only the self-adhesive resin cement and showed low bonding performance [28].

In this study, the specimens in group F showed the maximum bond strength regardless of the cyclic loading and abutment wall conditions. In the results for the fracture mode, groups C and A mainly showed Cc + Ai mixed fractures, while group F primarily showed Ri + Cc mixed fractures. In group F, the favorable dentin bonding achieved by the application of the resin adhesive system and the robust adhesion by the application of the flowable resin composite, which acts as a stress breaker, may have yielded a higher bond strength than that in group A, in which only the resin adhesive system was applied to the abutment dentin surface.

Human mastication produces a bite force of approximate 70–150 N [29,30], with approximately 60–90 cycles per minute [31,32]. The cyclic loading in this study was set at 78.5 N and 90 cycles per minute on the basis of these data for standard human mastication. The total number of 300,000 cyclic loadings is equivalent to a period of 14 months, based on an average of approximately 2.5 × 10^5^ mastication cycles per year [33]. However, the loading in this study was performed continuously. Therefore, cyclic loading without considerations for sleep and rest represents a more extreme experimental condition in comparison with the actual intraoral environment. Thus, severe cyclic loading can be considered to be a major factor that causes deterioration of the internal bonding of CAD/CAM ceramic crown restorations. Hayashi et al. [8] reported that the difference in the abutment wall condition of feldspathic ceramic CAD/CAM crown restorations applied for premolars does not influence the μTBS. Therefore, the differences between occlusal and axial wall conditions may not influence the quantitative internal bond strength of posterior CAD/CAM ceramic crown restorations, regardless of tooth kind (premolars or molars).

### 4.2. Differences in Weibull Parameters for the Occlusal and Axial Walls among the Three Types of Immediate Dentin Sealing with and without Cyclic Loading

In addition to quantitative evaluations based on µTBS, qualitative evaluations of the internal bonding state of CAD/CAM ceramic restorations were performed using Weibull analysis [22]. A high Weibull modulus is desirable for every material because it reflects greater homogeneity in defect distribution and greater predictability of failure behavior [34]. In fact, high Weibull moduli have been reported to indicate better bond reliability [35]. Furthermore, the ISO [23] states that the PF10 and PF90 can characterize the strength of a bond. In particular, the stress at the 10% probability of failure level, PF10, may be related to early-stage bonding durability because De Munck et al. [36] reported that the PF10 reflects early failures in the clinical situation.

In the present study, Wm, PF10, and PF90 in groups A and F, except in the S+/Ax/PF90 condition, were significantly greater than the corresponding values in group C (control), regardless of cyclic loading and abutment wall conditions. Moreover, the values in group C were the lowest in both conditions. Accordingly, the null hypothesis that the type of IDS does not influence the bonding of the CAD/CAM ceramic crown restorations, was rejected on the basis of quantitative and qualitative findings.

In group C, the bonding may have been solely based on the self-adhesive ability of the resin cement used in this study, yielding the formation of a hybrid layer with resin tags impregnated into the dentinal tubule. Notably, recent self-adhesive resin cements have not yet achieved dentin bonding performance similar to conventional adhesive resin cements using pretreatment agents [28,37]. Thus, the vulnerable bonding state in group C may be attributed to the poor forming ability of hybrid layers with resin tags [38] and complex chemical compositions [39]. Consequently, it was inferred that group A demonstrated a robust bonding state with favorable bonding reliability and durability because of the contribution of the hybrid layer generated by the all-in-one adhesive treatment.

Wm in group A was significantly greater than that in group F and yielded excellent bonding reliability, regardless of cyclic loading and abutment wall conditions. Robin et al. [34] described that a high Wm is desirable for an optimal bonding state. However, restoration with a higher Wm does not always offer a better clinical prognosis since a favorable clinical prognosis requires the combination of high bond strength, excellent bonding reliability with a high Wm, and superior bonding durability with large PF10/PF90. Although Wm in group A was the highest among the three IDS groups, μTBS and PF10/PF90 in group A were similar to or significantly smaller than in group F, regardless of cyclic loading and abutment wall conditions.

On the other hand, Breemer et al. [12] reported that a thin clinical IDS layer was vulnerable and could lead to the re-exposure of the cavity dentin surface with various treatment processes. In addition, De Goes et al. [40] examined the micro-tensile bond strength of adhesive systems to dentin with or without application of a flowable resin composite and reported the benefit of flowable resin layer at the point of improvement the marginal seal of the dentin tubules. Accordingly, it can be inferred that the bonding state of group A with a single application of the adhesive system may not reach the same level as in group F with combined application of both the adhesive system and flowable resin composite. The thick IDS layer formed using the flowable resin composite has been reported to act as a stress-breaker against cyclic loading and contributes to the improved bonding in comparison with the thin IDS layer [10], explaining why the cyclic loading-induced damage to the bonding interface in the F group was smaller than that in the A group.

Carvalho et al. [13] recommended clinical reinforcement of IDS with a flowable resin composite to achieve predictable bonding. Furthermore, Spohr et al. [41] stated that the film thickness formed by the use of adhesive and flowable resin composite as IDS materials may provide an increased fracture load and mentioned that an enhanced IDS layer may show better stress absorption against the shrinkage of resin cement. In this regard, the robustness of the IDS layer applied to the surface of the abutment is very important because the sealed dentin will be exposed to various forms of clinical stimulation until the completion of restoration. Consequently, IDS with the combined application of both an adhesive system and a flowable resin composite can be considered to be an excellent technique based on the results of both quantitative and qualitative investigations.

In assessments of the differences caused by cyclic loading conditions, cyclic loading clearly tended to cause deterioration of the qualitative bonding performance, regardless of IDS and abutment wall conditions. Therefore, the null hypothesis that the presence of cyclic loading does not affect the bonding was rejected on the basis of quantitative and qualitative findings.

These findings indicate that the use of severe cyclic loading simulating clinical mastication was an important factor that impaired not only quantitative solidity based on bond strength but also qualitative bonding reliability and durability.

In terms of the differences related to the abutment wall condition, the bonding reliability of the Ax wall tended to be superior to that of the Oc wall, and the bonding durability varied with cyclic loading and the abutment wall conditions. Accordingly, the null hypothesis that bonding does not differ between the occlusal and axial abutment walls was rejected on the basis of qualitative findings. With cyclic loading onto the occlusal surface of CAD/CAM ceramic crown restoration, the compressive stress mainly occurs on the Oc region of abutment, while the shear stress primarily occurs on the Ax region. Therefore, the differences in stress behavior on the abutment prepared dentin surface may reflect the differences in bonding reliability and durability between the Oc and Ax regions.

### 4.3. Fracture Mode Distribution of the Post-Bond Test Specimens

Cyclic loading showed the potential to damage the interface between resin cement and the abutment wall (Ai) or the resin cement itself (Cc) in groups C and A, while it attacked the interface between restorative and resin cement (Ri) or resin cement itself (Cc) in group F. Thus, in the F group, stress occurring at the interface between abutment dentin surface and resin cement was reduced by the flowable resin composite layer, which acted as an excellent stress breaker, unlike the findings in groups C and A, which were treated with only self-adhesive resin cement and cement with an all-in-one adhesive system, respectively.

Cc was observed on every fractured surface, regardless of IDS, cyclic loading, or abutment wall conditions. In assessments of flexural strength as an indicator of the substrate strength, the values of the restorative materials and dentin used in this study were as follows: ceramic block: 154 MPa [42], self-adhesive resin cement: 91 Mpa [43], flowable resin composite: 151 Mpa [44], and dentin: 213 Mpa [45]. Thus, resin cement, which showed the lowest flexural strength, had the potential to become a starting point for bonding failure of CAD/CAM ceramic crown restorations.

To resolve these failures through clinical manipulation, self-adhesive resin cements with greater strength are required. Moreover, a bonding interfacial zone consisting of a sealing layer combined with an adhesive system and a flowable resin composite (group F) is more robust than a bonding interface based on the self-adhesive ability of cement alone (group C) or the interface formed by single application of the adhesive system (group A).

The interfacial area in the three types of IDS may be strongly affected by cyclic loading. For the S+ condition simulating clinical mastication, the number of Ri + Cc mixed fractures in group F was obviously greater than those in groups C and A. On the other hand, the number of Cc + Ai mixed fractures in group F was clearly smaller than those in groups C and A. Accordingly, the bonding toughness of the abutment side in group F should be more robust than those of groups C and A, and the integration of abutment dentin and the self-adhesive resin cement in group F should be superior to those in groups C and A. In addition, if the bonding between the restorative and resin cement improves (e.g., by additional silane-coupling pretreatment onto the internal surface of CAD/CAM ceramic crown), the integration in group F would demonstrate a more stable and rugged state.

The surface structures of post micro-tensile bond test specimens were confirmed by the representative SEM images obtained in each condition. In particular, for the S+ condition, the presence of dentinal tubules was partially recognized on the surface of the specimens. In clinically poor situations such as the falling off of the CAD/CAM ceramic crown, patients may experience pain or hypersensitivity due to exposure of the dentinal tubules. The SEM images of groups A and F did not show exposure to the dentinal tubules and dentin surfaces regardless of cyclic loading. Therefore, the application of IDS performed by single application of an all-in-one adhesive system (group A) or combined application of both the adhesive system and a flowable resin composite (group F) may counteract the patient’s pain and hypersensitivity. This study was carried out under an in vitro experimental condition simulating the intra-oral environment. However, the condition did not sufficiently reproduce actual oral environments (e.g., food intervention, accurate mastication pattern, dynamic loading buffered with periodontal tissue). In addition, the cyclic loading in this study entailed 3 × 10^5^ cycles, and the number of the loading was equivalent to 14 months of human mastication. Therefore, the results obtained from this study were probably limited in the consideration of short-term clinical prognosis. Furthermore, combination-stress conditions with increased number of cyclic loading, thermal cycling, and pulpal pressure simultaneously are desirable in the future experimental design.

## 5. Conclusions

In the evaluation of the quantitative bonding performance of IDS, IDS performed with the single application of an all-in-one adhesive system (group A) and IDS with the combined application of the adhesive system and a flowable resin composite (group F) were significantly superior to the no IDS condition (group C), regardless of the cyclic loading and abutment wall conditions. In particular, group F showed the maximum bond strength. The qualitative bonding performances of IDS in groups A and F were superior to that in group C, regardless of the experimental conditions. In particular, for the cyclic loading condition simulating clinical mastication, the bonding durability in group F demonstrated the most outstanding state among the three IDS groups, regardless of the abutment wall conditions. Cyclic loading clearly tended to deteriorate quantitative and qualitative bonding performance, regardless of IDS and abutment wall conditions.

## Figures and Tables

**Table 1:** Materials used in this study.

Material	Composition	Lot Number	Manufacturer
Immediate Dentin Sealing Materials			
CLEARFIL Universal Bond Quick	MDP, Bis-GMA, HEMA, Hydrophilic amide monomers, Colloidal silica, Silane coupling agent, Sodium fluoride, Dl-camphorquinone, Ethanol, Water	870222	Kuraray Noritake Dental
CLEARFIL MAJESTY ES Flow	Barium grass filler, Silica filler, TEGDMA, Hydrophobic-aromatic dimethacrylate, Dl-camphorquinone, Photoinitiator	6D0238	Kuraray Noritake Dental
Adhesive resin cement system			
PANAVIA SA Cement Universal	Paste A MDP, Bis-GMA, TEGDMA, Hydrophobic aromatic dimethacrylate, HEMA, Silanated barium glass filler, Silanated colloidal silica, Dl-camphorquinone, Peroxide, Catalysts, Pigments Paste B Hydrophobic aromatic dimethacrylate, Silane coupling agent, Silanated barium glass filler, Aluminum oxide filler, Surface treated sodium fluoride, Dl-camphorquinone, Accelerators, Pigments	2K0054	Kuraray Noritake Dental
Pretreatment material for restorative			
Porcelain Etchant	Polyacrylamidomethylpropane sulfonic acid, Hydrofluoric acid	1800005815	Bisco
Additional building-up materials for micro-tensile bond strength			
CLEARFIL Universal Bond Quick	MDP, Bis-GMA, HEMA, Hydrophilic amide monomers, Colloidal silica, Silane coupling agent, Sodium fluoride, Dl-camphorquinone, Ethanol, Water	870222	Kuraray Noritake Dental
CLEARFIL AP-X	Bis-GMA, TEGDMA, Silanated barium glass filler, Silanated Silica filler, Silanated colloidal silica, Dl-camphorquinone	BK0132	Kuraray Noritake Dental
Chair-side CAD/CAM system			
CEREC AC Omnicam CEREC MC XL	CEREC operating system software version 4.5		Dentsply Sirona
Dental CAD/CAM restorative block			
VITABLOCS Mark II	Silicon dioxide, Aluminum oxide, Sodium oxide, Potassium oxide, Calcium oxide, Titanium dioxide	81550	VITA

MDP: 10-methacryloyloxydecyl dihydrogen phosphate, Bis-GMA: bisphenol A diglyciclmethacrylate, HEMA: 2-hydroxyethymethacrylate, TEGDMA: triethylene glycol dimethacrylate.

**Table 2:** Micro-tensile bond strength test results.

	Cyclic Loading	S−					S+				
Abutment Wall	Immediate Dentin Sealing	Median	Max/Min	Q1	Q3	ptf	Median	Max/Min	Q1	Q3	ptf
Oc	C	4.6	6.2/2.2	3.5	4.8	0	1.1	1.7/0.3	0.5	1.5	2
	A	8.2	9.2/6.6	7.2	8.8	0	2.2	2.6/1.9	1.9	2.4	0
	F	9.2	11.8/6.6	7.1	9.9	0	4.0	5.1/2.9	3.5	4.8	0
Ax	C	4.3	5.3/2.5	3.2	4.6	0	1.3	2.0/0.3	0.4	1.7	3
	A	8.1	9.0/7.1	7.5	8.7	0	2.1	2.6/1.5	1.9	2.2	2
	F	8.7	9.4/6.7	7.5	8.8	0	3.9	5.6/2.3	3.1	5.2	0

Q1: first quartile; Q3: third quartile; ptf: pretesting failure.

**Table 3:** Weibull parameter values of each experimental condition.

	Cyclic Loading	S−			S+		
Abutment Wall	Immediate Dentin Sealing	Wm	PF10	PF90	Wm	PF10	PF90
Oc	C	4.8	3.0 (2.7–3.2)	5.7 (5.4–6.1)	2.1	0.4 (0.4–0.5)	1.8 (1.7–2.0)
	A	11.0	6.9 (6.8–7.0)	9.1 (9.0–9.3)	9.9	1.9 (1.8–1.9)	2.5 (2.5–2.7)
	F	6.2	6.7 (6.3–7.0)	11.0 (10.6–11.5)	6.9	3.3 (3.1–3.4)	5.1 (4.9–5.3)
Ax	C	5.8	3.0 (2.9–3.2)	5.2 (5.0–5.4)	1.6	0.4 (0.2–0.5)	2.3 (1.8–3.4)
	A	14.4	7.2 (7.0–7.3)	8.9 (8.7–9.1)	9.1	1.7 (1.6–1.8)	2.4 (2.3–2.5)
	F	11.9	7.2 (7.1–7.3)	9.3 (9.2–9.5)	4.3	2.7 (2.5–2.8)	5.5 (5.2–5.9)

Wm: Weibull modulus; PF10: Weibull stress (MPa) for a 10% failure probability (95% confidence interval); PF90: Weibull stress (MPa) for a 90% failure probability (95% confidence interval).

**Table 4:** Distribution of fracture mode.

Cyclic Loading	S−						S+					
Abutment Wall	Oc			Ax			Oc			Ax		
Immediate Dentin Sealing	C	A	F	C	A	F	C	A	F	C	A	F
Ri + Cc	3	6	8	0	5	7	3	6	14	2	6	11
Cc + Ai	13	10	8	16	11	9	13	10	2	14	10	5
ptf (Fracture mode)	0	0	0	0	0	0	2 (Cc + Ai)	0	0	3 (Cc + Ai)	2 (Cc + Ai)	0

Ri: Interfacial fracture occurred at the interface between restorative and resin cement; Cc: Cohesive fracture occurred within the resin cement; Ai: Interfacial fracture occurred at the interface between resin cement and the abutment side surface (dentin, adhesive or flowable resin); ptf: Pretesting failure occurred during the trimming procedures.

## Data Availability

The data presented in this study are available on request from the corresponding author due to restrictions.
